# Complete mitochondrial genome of *Brachionus rubens* from wuhu, China (Rotifera, Brachionidae)

**DOI:** 10.1080/23802359.2021.1903351

**Published:** 2021-03-26

**Authors:** Yi Zhang, Hai Ying Zhang, Jin Jin Yan, Meng Ning Xia, Ying Long Liu, Yan Mei Lu, Yun Zhi Yan, Shuang Huai Cheng

**Affiliations:** a Public Research Lab of Hainan Medical University, Haikou, China; b Anhui Health College, Guichi, China; c Wuhu Aging Wise Biotechnology Co., Ltd, Wuhu, China; d State Key Laboratory of Freshwater Ecology and Biotechnology, Institute of Hydrobiology, Chinese Academy of Sciences, Wuhan, China

**Keywords:** Rotifera, *Brachionus rubens*, *Brachionus*, mitochondrial genome

## Abstract

The complete mitochondrial genome of *Brachionus rubens* was sequenced using primers design, clone culture, DNA extraction, LONG-PCR amplification, purification and clone sequencing. We found that it is composed of two circular chromosomes, designated mtDNA I (11,398 bp) and mtDNA II (12,820 bp). The gene content of the *B. rubens* mitochondrial genome was similar to that of the previously reported mitochondrial genome of *B. plicatilis*. It contained 22 tRNA genes, 2 rRNA genes and 12 protein-coding genes (PCGs). Four of the 12 PCGs had an incomplete stop codons, TA（cob, atp6, nd3）or T(cox3). The A + T content of *B. rubens* mitochondrial genome was apparently higher (mtDNA-I 70.2% and mtDNA II 70.4%) than that of the mitochondrial genome of *B. plicatilis* (mtDNA-I 63.9% and mtDNA-II 62.9%).

To date, only a few complete mitochondrial genomes have been published for freshwater rotifers: *Brachionus calyciflorus* (Nie et al. 2016; Choi et al. 2019) and *B. rubens* from Japan (Choi, Lee, et al. 2020). Several mitogenomes of marine rotifer *Brachionus sp*. were previously reported (Suga et al. 2008 for *B. plicatilis*; Hwang et al. 2014 for *B. koreanus*, Kim et al. 2017 for *B. rotundiformis* and Choi, Kin, et al. 2020 for *B. paranguensis*). *Brachionus rubens* is an important, globally distributed freshwater rotifer that has been used in environmental toxicology studies to investigate its response to temperature and metal ion concentration (Azuara-García et al. 2006; Montúfar-Meléendez et al. 2007), toxic cyanobacteria (Geng et al. 2006; Geng and Xie 2008; Pérez-Morales et al. 2014). The results of these studies suggested that *B. rubens* is an important member of freshwater zooplankton communities and a good candidate species for ecotoxicology tests. The analysis of the mitochondrial genome of *B. rubens* is important for identifying field-samples and laboratory stocks and may also be important to address questions in relation to the genetic structure of populations. In this study, we sequenced the complete mitochondrial genome of the monogonont rotifer *B. rubens* from a population in China and analyzed its phylogenetic placement in relation to other freshwater rotifers in the *Brachionus* clade.

The samples of *B. rubens* were collected from the Changjiang river, Wu Hu segment (Longitude: 31.20, Latitude: 118.21) using a 30-μm plankton net, and maintained at our lab in Anhui Normal University in China (catalog number: BRWH001). They were individually cultured in the lab at 18 °C (similar to the water temperature when the rotifers were collected) and fed daily on *Scenedesmus obliquus* following the technique detailed in Cheng and Xi (2009). Twelve of specimens were successfully clonally cultured. To avoid the contamination of rotifer genomes with food organisms, live *B. rubens* were starved for one day before harvesting. As they reached higher densities (200–300 individuals mL^−1^), the rotifers were filtered with a 30-μm plankton net, washed several times with double distilled water, centrifuged, and preserved in the freezer until molecular processing. Total genomic DNA was isolated using the Wizard^TM^ genomic DNA purification kit, as described by Dong et al. (2002). PCR amplifications were performed in a thermo-cycler using 10 pairs of primes to amplify different regions of mt DNA with overlaps in the same region.

The complete mitochondrial genome of *B. rubens* were 11,398 bp (mitochondrial DNA I; Genbank no. KJ489417) and 12,820 bp (mitochondrial DNA II; Genbank no. KJ489418) in size. The direction of the 12 protein-coding genes (PGCs) of *B. rubens* from China was identical to those of *B. rubens* from Japan, including the presence of a nearly identical non-coding region (identity: 99.5%) (Suga et al. 2008; Hwang et al. 2014). Of the 12 PCGs, one gene is initiated by ATA (nad4), and one gene (cox2), was determined to begin with ATT, while the start codon of the other PCGs was ATG. Eight protein-coding genes terminated with the complete stop codons TAA (cox1, cox2, nad5, nad6, nad2 and nad4L) or TAG (nad1, nad4). The other four genes had incomplete stop codons: TA– (nad3, cob, and atp6) and T–(cox3). In both mt DNA chromosomes, T is strongly favored over A, G, and C, at third codon positions, 60.66%, 23.89%, 10.14% and 5.3%, respectively. The most common amino acids encoded by the *B. rubens* mitochondrial genes are leucine (16.7%) and serine (11.01%), which are also the only amino acids with 2 tRNAs.

The placement of *B. rubens* from China in the genus *Brachionus* with 12 PCGs nucleotide sequences is shown in Figure 1. The *B. rubens* sequence from Japan clustered with *B. rubens* from China. The gene order and contents of 12 PGCs were identical to those of the freshwater rotifer *B. rubens* Japan. The order of tRNAs of three tRNA-Leu, tRNA-Ile, and tRNA-Arg was different between both *B. rubens* mitochondrial chromosomes. This suggests that different geographic strains of the same species maybe have different gene arrangements.

**Figure 1.**
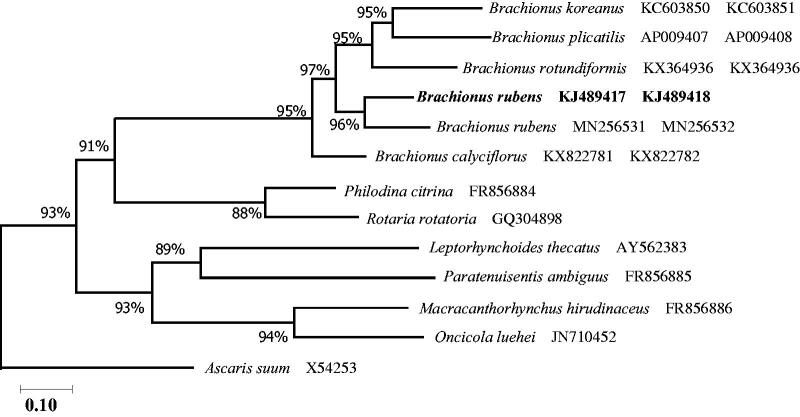
Phylogenetic analysis of 12 PCGs nucleotide sequences from the rotifer *B. rubens*. The evolutionary history was inferred by using the maximum likelihood method and Tamura–Nei model (Tamura and Nei 1993). The tree with the highest log likelihood (–124,812.25) is shown. Initial tree(s) for the heuristic search were obtained automatically by applying the Neighbor-Joining and BioNJ algorithms to a matrix of pairwise distances estimated using the Tamura–Nei model, and then selecting the topology with superior log likelihood value. The tree is drawn to scale, with branch lengths measured in the number of substitutions per site. The proportion of sites where at least 1 unambiguous base is present in at least 1 sequence for each descendent clade is shown next to each internal node in the tree. This analysis involved 13 nucleotide sequences. Codon positions included were 1st + 2nd + 3rd + Noncoding. There were a total of 11,032 positions in the final dataset. Evolutionary analyses were conducted in MEGA X (Kumar et al. 2018).

## Data Availability

The authors confirm that the data supporting the findings of this study are available within the article or its supplementary materials. http://purl.org/phylo/treebase/phylows/study/TB2:S27164?x-access-code=ce99974bb4701c6182774ecc3e3cde6c&format=html; https://www.ncbi.nlm.nih.gov/nuccore/KJ489417; https://www.ncbi.nlm.nih.gov/nuccore/KJ489418.
